# Supplementary material for: Membrane Lipid Requirements of the Lysine Transporter Lyp1 from Saccharomyces cerevisiae
Source: J Mol Biol. Author manuscript; Available in PMC 2021 Mar 29. (PMC8005870; doi:10.1016/j.jmb.2020.04.029)
Supplement: Figures S1-S3 [file NIHMS1681749-supplement-Figures_S1-S3.pdf]

SUPPLEMENTARY INFORMATION

Membrane lipid requirements of the lysine transporter Lyp1 from *Saccharomyces cerevisiae*

Joury S van 't Klooster[a], Tan-Yun Cheng[b], Hendrik R Sikkema[a], Aike Jeucken[a], D. Branch Moody[b,c] and Bert Poolman[a,*]

[a]Department of Biochemistry, University of Groningen
Groningen Biomolecular Sciences and Biotechnology Institute
Nijenborgh 4, 9747 AG Groningen, The Netherlands

[b]Division of Rheumatology, Immunology and Allergy
Brigham and Women's Hospital, Harvard Medical School
60 Fenwood Road, Boston, MA, 02115, USA.
[c]Department of Medicine, Harvard Medical School, Boston, MA, 02115, USA.

[*]To whom correspondence should be addressed
E-mail: b.poolman@rug.nl or bmoody@bwh.harvard.edu

Supplementary Figures S1-S3.

Supplementary figures

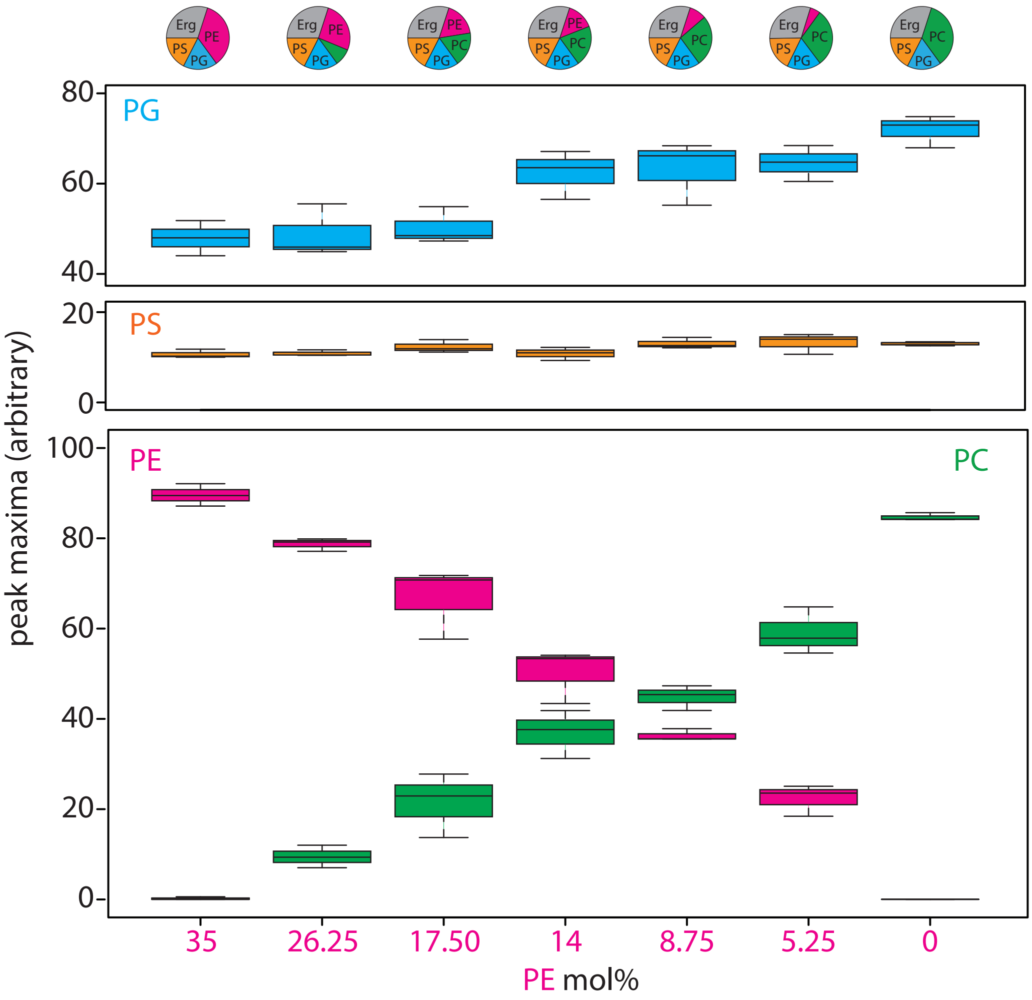

Figure S1: Lipid analysis by mass spectrometry of proteo-liposomes. Boxplots show peak areas of mass spectra corresponding to POPE, POPG, POPS and POPC, present in in respective Lyp1 vesicles. For POPG and POPS the data was recorded in negative mode, for POPE and POPC data was recorded in positive mode. The peak maxima of POPG drift from a minimum of 50 to a maximum 70 au, which results in POPG values ranging from 17.5% +/- 3.0%. We have no explanation for the larger variation in PG values as compared to those of the other lipids. Absolute quantities of lipids (mol%) taken for each lipid composition are shown in the pie-graphs above. Two-letter abbreviation and color-coding: PC = phosphatidylcholine (POPC) (green), PE = Phosphatidylethanolamine (POPE) (magenta), PS = phosphatidylserine (POPS) (orange) and PG = phosphatidylglycerol (POPG) (blue). Line within boxplot represents the median. Top and bottom represent the first and third quartile, respectively. Error bars are the minimal and maximal value. Number of experiments = 3 experimental replicates.

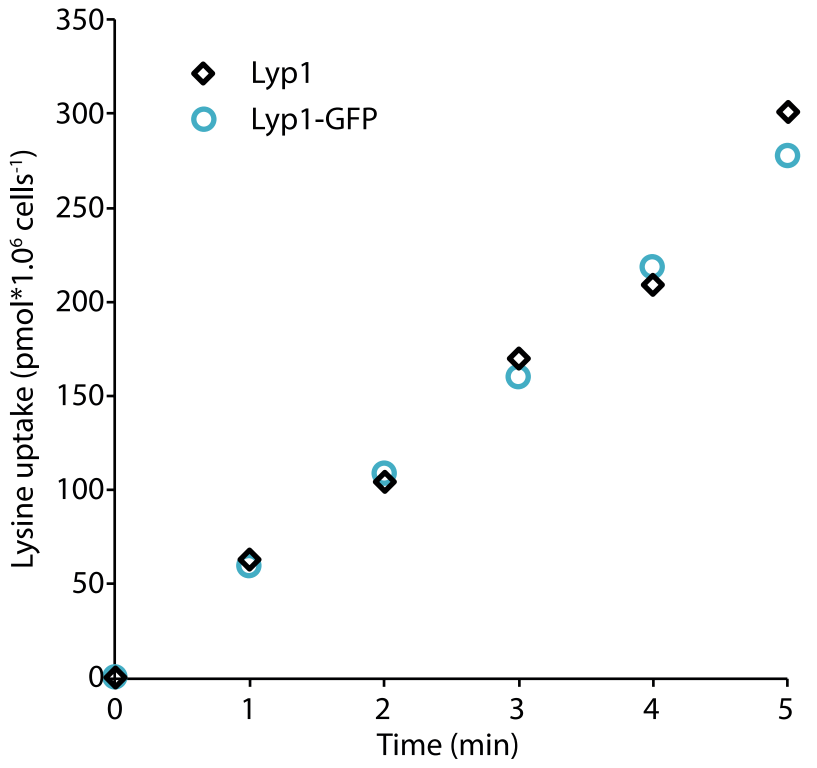

Figure S2. Lysine transport by Lyp1 and Lyp1-GFP in *S. cerevisiae* BY4742, carrying the expression vector pFB021. Transport of lysine at pH 6.0 at an external [^{14}C]-lysine concentration of 20 M as described in[32]

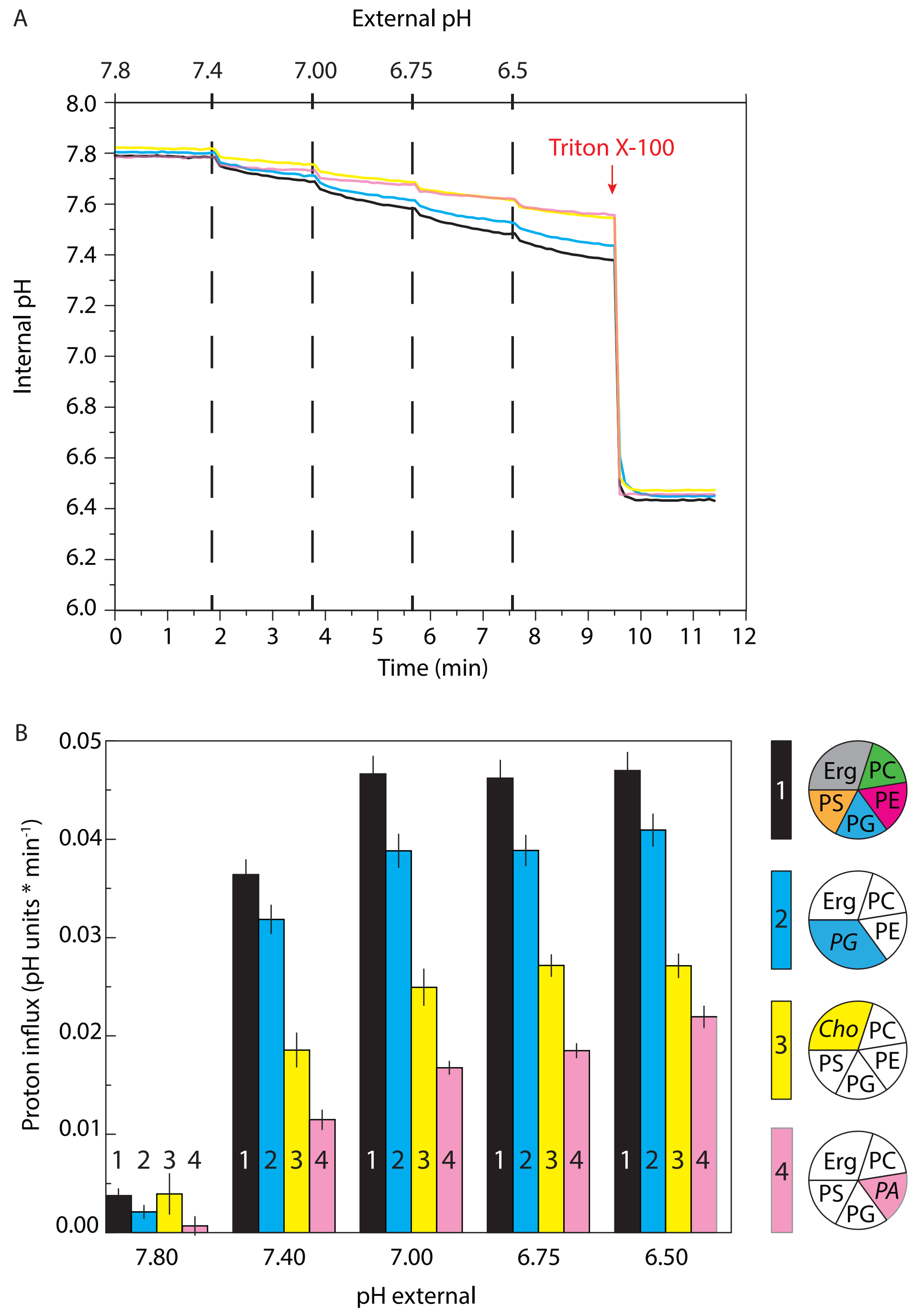

Figure S3. Proton permeability of proteoliposomes. (A) Internal pH is shown as a function of external pH and time for proteoliposomes composed of various lipids. The vesicles were prepared in 10 mM potassium-phosphate pH 7.8. Color code is: POPC/POPE/POPG/POPS/Ergosterol (17,5/17,5/17,5/17.5/30 mol%) = black; POPC/POPE/POPG/Ergosterol (17,5/17,5/35/30 mol%) = Blue; POPC/POPE/POPG/POPS/Cholesterol (17,5/17,5/17,5/17.5/30 mol%) = Yellow; POPC/POPG/POPS/POPA/Ergosterol (17,5/17,5/17,5/17.5/30 mol%) = Pink. The red-arrow indicates Triton X-100 addition, which solubilizes the proteoliposomes and releases pyranine. (B) Proton influx (pH units * min^{-1}) is shown as a function of external pH for each lipid composition. The proton influx was calculated from the slope of a linear fit of the data, where dashed lines in Panel A indicate the start and end points used for the fit. Result is representative of three experiments, and error bars are the standard error of the fit.
